# A Comparison of Loop-Mediated Isothermal Amplification (LAMP) with Other Surveillance Tools for *Echinococcus granulosus* Diagnosis in Canine Definitive Hosts

**DOI:** 10.1371/journal.pone.0100877

**Published:** 2014-07-09

**Authors:** Xing-Wei Ni, Donald P. McManus, Zhong-Zi Lou, Ji-Fei Yang, Hong-Bin Yan, Li Li, Hong-Min Li, Quan-Yuan Liu, Chun-Hua Li, Wan-Gui Shi, Yan-Lei Fan, Xu Liu, Jin-Zhong Cai, Meng-Tong Lei, Bao-Quan Fu, Yu-Rong Yang, Wan-Zhong Jia

**Affiliations:** 1 State Key Laboratory of Veterinary Etiological Biology/Key Laboratory of Veterinary Parasitology of Gansu Province/Key Laboratory of Zoonoses of Agriculture Ministry/Lanzhou Veterinary Research Institute, Chinese Academy of Agricultural Sciences, Lanzhou, Gansu Province, P. R. China; 2 Molecular Parasitology Laboratory, Queensland Institute of Medical Research, Brisbane, Queensland, Australia; 3 Gansu Provincial Center for Animal Disease Control and Prevention, Lanzhou, Gansu Province, P. R. China; 4 Qinghai Academy of Animal Science and Veterinary Medicine, Xining, Qinghai Province, P. R. China; 5 Ningxia Medical University, Yinchuan, Ningxia Hui Autonomous Region, P. R. China; GI Lab, United States of America

## Abstract

**Background:**

Cystic echinococcosis is highly prevalent in northwest China. A cost-effective, easy to operate diagnostic tool with high sensitivity and specificity would greatly facilitate the monitoring of *Echinococcus* infections in canine definitive hosts.

**Methods:**

The primers used in the LAMP assay were based on the mitochondrial *nad*5 gene of *E. granulosus sensu stricto* (*E. granulosus s.s.*, or *E.g.s.s.*) and were designed using Primer Explorer V4 software. The developed LAMP assay was compared with a conventional PCR method, copro-ELISA and microscopy, using the faeces of dogs experimentally infected with *E.g.s.s.*, and field-collected faeces of domestic dogs including 190 from Qinghai province highly endemic for *E.g.s.s.* and 30 controls from an area in Gansu, where a domestic dog de-worming program was in operation.

**Results:**

The positivity rates obtained for the field-collected faecal samples were 12.6%, 1.6% and 2.1% by the LAMP, PCR and copro-ELISA assays, respectively. All samples obtained from the control dogs were negative. Compared with the conventional PCR, the LAMP assay provided 88.8% specificity and 100% sensitivity. The higher sensitivity of the LAMP method was also shown by the fact that it could detect the presence of laboratory challenge dog infections of *E. granulsous s.s.* four days earlier than the PCR method. Three copro-samples shown positive by the commercial copro-ELISA were all negative by LAMP, PCR and microscopy, which suggests these samples may have originated from another infection rather than *E. granulsous s.s.*, possibly *E. shiquicus* or *E. Canadensis,* which is also present in China.

**Conclusions:**

We have developed a potentially useful surveillance tool for determining the prevalence of canine *E. granulosus s.s.* infections in the field. The LAMP assay may lead to a more cost-effective and practicable way of tracking *Echinococcus* infections in canids, especially when combined with the copro-ELISA.

## Introduction

Cystic echinococcosis (CE), caused by *Echinococcus granulosus* (*E.g.*), is of considerable importance from the public health perspective and also has a significant socio-economic impact. There are an estimated 2–3 million cases of human CE [1], [2] with its global burden in disability-adjusted life years (DALYs) estimated in 2006 to be 285,000 with an annual economic loss of US$ 194 million [3]. The most recent (2010) DALY measure for echinococcosis was reported to be 144,000 [4]. The World Health Organization has included echinococcosis as one of a group of zoonoses for its 2008–2015 strategic plan for the control of neglected tropical diseases (NTDs) [5], due to its widespread distribution in both developing and developed countries [6].

An important feature of the biology of *E.g.* is the fact that it comprises a number of intra-specific variants or strains that exhibit considerable variation at the genetic level [7]. *E.g. sensu lato* (*E.g.s.l.*) comprises previous 10 genotypes (G1 to G10) with the G1 (sheep strain) genotype being the prototypical species, infecting humans and livestock extensively [8]. The G1 (named *E. granulosus sensu stricto*, *E.g.s.s.*) and G6 (camel/dog strain, named *E. canadensis*) genotypes have been reported within China [9]. The definitive hosts of *E.g.* are canids - dogs, dingoes, foxes, wolves and jackals - which harbour the adult stage in the small intestine. Eggs and gravid proglottides are released periodically in faeces, into the external environment. Humans and herbivorous animals become infected with the metacestode (larval stage) of *E.g.* following the ingestion of viable eggs. Transmission is completed when the viscera of infected intermediate host animals (generally slaughtered livestock) are consumed by canids [10], which in turn transmit CE infection to livestock and humans. Consequently, in order to effectively control the transmission of *E.g.*, efforts should be directed towards building an effective surveillance system for identifying infected canine hosts, an important component for establishing the epidemiological parameters of CE and for preventing human and livestock infection [11]. However, the detection of *E.g.* in canids is difficult as the infection is generally asymptomatic and the small proglottides spontaneously discharged in faeces are usually overlooked [12]. Furthermore, routine copro-microscopy examination cannot differentiate the eggs of *E.g.* from other *Taenia* species [12] and, although extensively used, purgation with arecoline compounds and necropsy of the small intestine [12], [13], is laborious and time-consuming and is impractical for large-scale surveys.

The application of immunological approaches and polymerase chain reaction (PCR)-based procedures has proven of value in the detection of *E.g.* infection in definitive hosts using defined parasite copro-antigens or DNA sequences [12], although challenges remain in terms of sensitivity and specificity [10]. Further, for routine laboratory diagnosis and surveillance, DNA methods have a considerable drawback, in that the sensitivity of conventional PCR can be severely affected by inhibitory factors present in faecal samples [14]. The expensive facilities and reagents and the relatively long time required for test completion are additional disadvantages [15], [16]. Accordingly, we have developed an effective surveillance tool based on loop-mediated isothermal amplification (LAMP) for the detection of *E.g.*-positive canine faecal samples collected in the field. LAMP-based assays [17], [18] are more convenient and affordable than other traditional surveillance tools and they can lead to the more rapid detection of infected canines. Rapid, simple, specific and sensitive LAMP assays, have been described for the coprodetection of *E.g.* infections [19], [20]. However, there are no reports, to date, of the use of LAMP as a tool for the detection of *E.g.* in faecal samples from naturally infected dogs. Here we describe the development of an alternative LAMP assay, based on detecting a specific partial sequence of the mitochondrial *nad*5 gene of *E.g.s.s.* in the faeces of dogs experimentally infected with metacestodes of the *E.g.s.s.* We have assessed its value on canine faecal samples collected in the field and compared its practicability and diagnostic performance with conventional PCR, ELISA and microscopy approaches.

## Materials and Methods

### Ethics statement

The institutional ethical committee of Lanzhou Veterinary Research Institute, Chinese Academy of Agricultural Sciences, Lanzhou, Gansu Province approved the study (Approval No. LVRIAEC 2010-005). The experiments using dogs were undertaken under very strict adherence to the institutional and Chinese national guidelines for animal husbandry. For any locations and activities in the field studies, no specific permissions were required, or no endangered/protected species were involved in.

### *E. granulosus s.s.* materials

An *E.g.s.s.* isolate [21] [confirmed subsequently by *cox*1 and *nad*1 sequencing (data not shown)] was obtained from a large unilocular hydatid cyst of *E.g.s.s.* in a sheep liver at an abattoir in Xining city, Qinghai province in 2010. The hydatid fluid was aspirated into a 500-ml conical flask and protoscoleces were obtained by sedimentation. The sediment was washed four times with sterilized phosphate-buffered saline (PBS). The protoscoleces were then immediately immersed in Dulbecco's Modified Eagle Media (DMEM) containing 5% (v/v) fetal bovine serum (FBS) at 37°C and maintained for two hours in order to determine their viability by observing their movement microscopically. Samples having 95% viable protoscoleces were used for challenging dogs or stored at −70°C for DNA isolation.

### Experimental infection of dogs

Four 6-month old non-pedigree dogs were purchased from a dog-market in Lanzhou city, Gansu province. They were treated with praziquantel (10 mg/kg body weight given on three consecutive days per month) for two months prior to the study commencement, and kept in individual cages at the experimental facility, Lanzhou Veterinary Research Institute, to allow them to adapt to the living environment and diet. After verification that they were helminth worm-free, by microscopic examination of their faeces, each dog (average weight 10 kg) received orally about 10,000 viable protoscoleces of the *E.g.s.s.* isolate with a meal, and then they were fed a heat-treated meal once daily. Faecal samples were collected daily and examined carefully for the *E.g.* proglottides with macroscopic and stereo microscopic observation after sufficiently homogenizing, until day 70 post challenge, from the bottom of individual cages. They were placed into sterilized 50-ml containers with tight fitting lids and stored at −70°C. The dogs were euthanized to check the small intestine for the determination of the number of the *E.g.s.s.* worms on day 76 post-challenge infection.

### Field collection of copro-samples from naturally infected domestic dogs

Faecal samples (n = 190) were collected from individual domestic dogs in two *E.g.*-endemic areas; Zhiduo county (N33°36′1.08″, E96°03′50.04″ to N33°48′31.47″, E95°25′22.48″; altitude of approximately 4300 m) and Dari county (N33°43′38.24″, E99°25′50.51 to N33°39′45.93″, E98°59′27.58″; altitude of 4000∼4300 m), Qinghai province [22]. Negative control dog faeces (n = 30) were collected from Tianzhu county (N37°11′13.90″, E102°48′3.27″; altitude of approximately 2900 m), Gansu province, where mass dog treatment with praziquantel (10 mg/kg) had been previously carried out monthly for more than one and a half years. All collected faecal samples were stored at −70°C before use for microscopic examination or for isolation of genomic DNA and copro-antigens.

### Microscopic examination for the presence of taeniid eggs

Faecal samples from all naturally infected dogs were fully homogenized and then subjected to a conventional saturated sodium chloride (NaCl) flotation method [23]. Briefly, 2 g faeces were washed with distilled water and sedimented by centrifugation at 2,500×g for 10 min. The supernatant was collected, aliquotted into tubes and stored at −20°C before being used for copro-ELISA detection. The sediment was suspended in saturated NaCl solution and any eggs present were observed by light microscopy and recorded. All the copro-samples, including those either egg-positive or egg-negative, were further tested by the LAMP and PCR assays.

### Parasite and host DNA extraction

Genomic DNA (g-DNA), obtained from protoscoleces of the G1 isolate using an Axyprep multisource genomic DNA miniprep kit (Axygen, CA, USA), acted as an *E.g.s.s.*-positive control to assess the sensitivity of the LAMP assay. *E.g.s.s.* genomic DNA isolated from faecal samples (f-DNA) was obtained from the faeces of the experimentally infected dogs and faeces from naturally infected domestic dogs. Parasite DNA (f-DNA) was extracted from the faecal samples (200 mg) using Axyprep kits and QIAamp DNA stool mini kits (Qiagen, Germany). Genomic DNA samples (g-DNAs) from *E.g.s.l.* including G4 (named *E. equinus*), G6 and G6/G7 genotypes (named *E. canadensis*), *E. multilocularis*, *E. shiquicus*, *T. hydatigena*, *T. pisiformis*, *T. taeniaeformis*, *T. multiceps* and *Dipylidium* sp. were used to determine the specificity of the *E.g.s.s.* LAMP assay. The *T. taeniaeformis* g-DNA was provided by Viktor Dyachenko, Institute for Infectious Diseases and Zoonoses, Ludwig-Maximilians-University of Munich, Munich, Germany, the *E. shiquicus* g-DNA was extracted from a cyst collected from a naturally infected plateau pika (*Onchotona curzoniae*) from Shiqu (N33°09′54.04″, E97°32′59.94″; altitude of approximately 4500 m) in 2011, and the g-DNAs of the *E. equinus* and *E. canadensis* isolates were provided by Antonio Varcasia from Dipartimento di Biologia Animale, Università degli Studi di Sassari, Italy. The other cestode g-DNAs, isolated using Axyprep kits, were obtained from worms from experimentally infected dogs at Lanzhou Veterinary Research Institute. In addition, intestinal contents (200 mg) and negative faecal samples (200 mg) (n-f-DNA) from uninfected dogs were obtained from newly born pups and the DNAs were extracted (Axyprep kits) to serve as negative controls. The concentrations of the DNA samples were measured using a Nanodrop 2000 spectrophotometer (Thermo Scientific, China).

### Conventional PCR assay

A conventional PCR assay was carried out for comparative purposes. The PCR primers (Eg1f: 5′-CAT TAA TGT ATT TTG TAA AGT TG-3′; Eg1r: 5′-CAC ATC ATC TTA CAA TAA CAC C-3′) were used to amplify a fragment of the mitochondrial 12S rRNA gene of *E.g.* [24]. The PCR reactions were performed according to Štefanić et al. [24] as follows: 50 µl PCR-mixture comprising 10 mM Tris-HCl (pH 9), 50 mM KC1, 2 mM MgCl2, 200 µM of each dNTP, 0.2 µM each primer, 1.25 U Taq polymerase (TaKaRa, Dalian, China) and 1 µl of DNA sample. The thermal cycling conditions comprised incubation at 95°C for 4 min; 35 cycles at 94°C for 30 s, 53°C for 30 s and 72°C for 30 s with a final extension at 72°C for 10 min.

### LAMP assay

LAMP primers were designed based on the amplification of a specific sequence within the mt *nad*5 gene of *E.g.s.s.* (GenBank accession no. AF297617 or NC_008075) [21] using Primer Explorer V4 software (http://primerexplorer.jp/elamp4.0.0/index.html). The sequences for the F3 and B3 primers are located outside of the two other primers (FIP and BIP) in the *E.g.s.s.* mt-*nad*5 gene region. Primers were validated using BLAST software (http://www.ncbi.nlm.nih.gov/BLAST), and their sequences are listed in Table 1. The LAMP reaction was performed in a 25 µl volume with 2 µl of target sample, 1.8 µl of primer mix (40 pmol each of FIP and BIP, 5 pmol each of F3 and B3), 1.0 µl of Bst DNA polymerase (8 U), 2.5 µl of 10×reaction buffer, 0.5 µl of 25 mM dNTPs, 5 µl of 5 M betaine, 1 µl of 100 mM MgSO4, and 11.2 µl of ddH2O.

**Table 1:** LAMP primers (Patent application No. 201110346441) targeting the mitochondrial *nad*5 gene of *E. granulosus* G1 genotype.

Name	Sequence (5' → 3')
FIP	TAACCCAAACGTTACCACATCAAAAgaattcTAGATTTTTGTCTACTATGGGTTGT
BIP	ATACTGGTCATTATTTCGGCGGgaattcCAAACAAAACAATCAACTTCAAC
F3	TGGTTTTAGGTATTTGATTAGGT
B3	ACCACTACATACCAACACC

Note: The lower case italicized gaattc in the primers FIP and BIP shows the position of the introduced *Eco*RI restriction site.

To determine the optimal reaction temperature and time, the reaction mixture was incubated at 60°C, 61°C, 62°C, 63°C, 64°C and 65°C, respectively, for 30 min, then heated at 80°C for 5 min to terminate the reaction; then six different reaction time periods (10, 20, 30, 40, 50 and 60 min) were compared at the optimal reaction temperature.

### The specificity and sensitivity of the LAMP assay

To verify the specificity of the LAMP assay for detection of *E.g.s.s.* DNA, the LAMP primers were tested using g-DNAs from other *E.g.s.l.*, *E. multilocularis*, *E. shiquicus*, *T. hydatigena*, *T. pisiformis*, *T. taeniaeformis*, *T. multiceps*, *D. caninum*, n-f-DNA (faecal samples from cestode-free dogs) and dog intestinal tissue as negative controls. To further confirm the specificity of the LAMP amplifications, the sequences of the LAMP amplicons were determined using a modification of the method described by Nkouawa et al [18]. Briefly, the LAMP products were digested at 37°C with EcoRI (TaKaRa, Dalian, China) for four hours. The digested products, purified using Axyprep DNA Gel Extraction Kits, were ligated into pMD-18 T vector at 4°C overnight. The ligation mixtures were used to transform Escherichia coli JM109 cells by incubating for 12 h at 37°C [18]. Single colonies were cultivated in Luria-Bertani medium (LB) with Amp + at 37°C for 12 h and then were analyzed by PCR using vector primers (M13F/M13R). Positive colonies with a PCR-amplified fragment (∼200 bp) of *E.g.s.s.* mt-*nad*5 were sequenced by Sangon Biotech Co., Ltd. (Shanghai, China). In order to determine the sensitivity of the LAMP assay, *E.g.s.s.* g-DNA was diluted to 10 ng/µl, then successively diluted 10 times by the addition of 1 µl of a 1/10 dilution of the previous concentration. The same dilution procedure was also performed on DNA samples from dog faeces (f-g-DNA) obtained at different days post-*E.g.s.s.* metacestode challenge. In addition, the minimum number of eggs detected by the LAMP assay was determined in the experiments with faeces spiked with eggs obtained from *E.g.s.s.* adults collected from one of the experimentally infected dogs. The eggs were counted, mixed with faeces from an uninfected dog and the faecal samples were then frozen until use.

### LAMP/PCR analysis of field-obtained dog faecal samples

The 190 field-obtained faecal samples collected from dogs, which included samples with taeniid eggs present confirmed by microscopy, were all subjected to the LAMP and PCR assays. The f-g-DNA extracted from the faeces of one of the dogs experimentally infected with *E.g.s.s.* was used as positive control. The LAMP and PCR products were electrophoresed on a 1.5% (w/v) agarose gel with ethidium bromide and photographed using a gel documentation system. Also, the LAMP products were characterized by visual inspection after the addition of a 1/10 dilution of 1/10000 concentration SYBR Green I (Invitrogen) to the reaction tube.

### Copro-ELISA analysis of field-obtained dog faecal samples

Two grams of faecal sample were mixed (1∶2) with phosphate buffered saline (pH 7.2) containing 0.3% (v/v) Tween 20 (PBS-T) in a 15 ml centrifuge tube at room temperature; the tube was shaken vigorously and the contents allowed to sediment. The supernatant was collected into a 2 ml screw capped tube, labelled with a reference number and stored at −20°C until analyzed using a commercial copro-antigen sandwich ELISA kit (Zhuhai S.E.Z Haitai Biological Pharmaceuticals Co., Ltd., Zhuhai, China) according to the manufacturer's instructions. One hundred µl of faecal supernatant in 0.15 M PBS-T was added to the wells of polystyrene plates that had been coated with specific antibody prepared against *E.g.* antigens (antigen components undisclosed by the makers of the commercial kit) and incubated at 37°C for one hour. One positive, one negative and three cut-off controls, provided in the kit, were placed in wells of each plate. The plates were washed three times (3 × 3 min) with washing buffer (0.05% PBS-Tween 20); then, 50 µl anti-*E.g.* specific antibodies conjugated with horseradish peroxidase (HRP) (provided in the kit) were added to each well and incubated at 37°C for 30 min. The wells were washed three times in the washing buffer, 50 µl each of colour reagent A and B (provided in the kit with 3,3′,5,5′- tetramethylbenzidine, TMB) were added, the solution was incubated at 37°C for 30 min in the dark and then the reaction was stopped with 50 µl stop solution. Absorbance values were read on a Multiscan ELISA reader at 450 nm. The sample-positive OD-value was used as the average OD-value of the three cut-off controls. The sensitivity and specificity of the ELISA was determined using the conventional PCR assay as reference standard.

### Statistical analysis

Differences among the LAMP, PCR and ELISA procedures and microscopy for assessing the sensitivity of each test were determined using One-Way ANOVA with post hoc LSD tests and the Chi-square test using the software package SPSS 11.5 [25].

## Results

### Optimal reaction temperature and time for the LAMP assay

**Figure 1:**
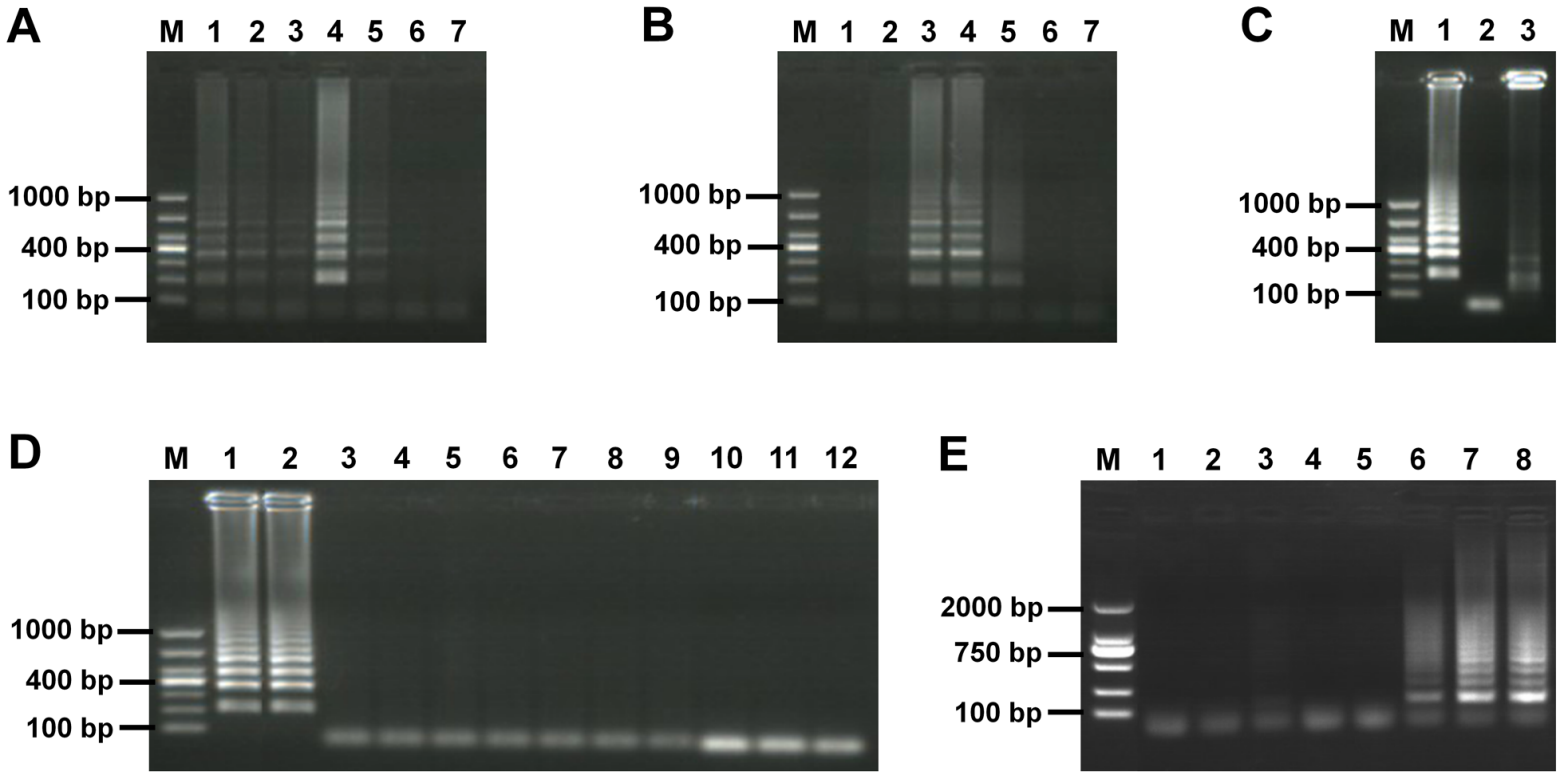
Establishment of the LAMP assay. A. Different reaction temperature. Lanes: M, DNA marker; 1 to 6, 60 to 65°C; 7, water control. B. Different reaction time. Lanes: M, DNA marker; 1 to 6, 10 to 60 min; 7, water control. C. Amplification of LAMP from positive f-DNA and restriction digestion of LAMP product. Lanes: M, DNA marker; 1, LAMP products from positive f-DNA; 2, water control; 3, *Eco*R1 digestion of LAMP products. D. Specificity of LAMP assay for *E. granulosus* (G1 genotype). Lanes: M, DNA marker; 1 and 2, g-DNA and f-DNA of *E. granulosus* (G1 genotype); 3 to 9, g-DNA of *E. multilocularis*, *E. shiquicus*, *T. hydatigena*, *T. multiceps*, *T. pisiformis*, *T. taeniaeformis*, *Dipylidium* sp.; 10, intestinal tissues of dogs; 11, n-f-DNA; 12, water control. E. Sensitivity of LAMP assay for DNA from different numbers of *E. granulosus* eggs per gram of faeces. Lanes: 1, negative control; 2 to 6, one to five eggs; 7, ten eggs; 8, fifteen eggs; M, DL2000.

Of a range of temperatures tested, 63°C was chosen as the optimal reaction temperature (Fig. 1A). The optimal length of time for the assay reaction, determined using a range from 10 to 60 min tested at 63°C, indicated 30 to 40 min was optimal (Fig. 1B). Accordingly, 40 min was chosen for all subsequent assays.

### Analysis of the digested LAMP products

The LAMP products demonstrated typical patterns of ladder-like bands on agar gels, and their EcoRI digestion products were as expected (Fig. 1C). The LAMP products with the correct target gene and sequence were confirmed by DNA sequencing (data not shown).

### Experimentally infected dogs

All six chellanged dogs were euthanized to retrieve the dogs' intestines for the confirmation of the *E.g.s.s.* infections. Four of the six dogs yielded 420, 321, 302 and 119 *E.g.s.s.* worms; no worms were recovered from the other two dogs.

### The specificity and sensitivity of the LAMP assay

*E.g.s.s.* g-DNAs and f-g-DNA, as well as g-DNAs extracted from the other parasites and dog host intestinal tissues were tested to determine the specificity of the LAMP assay for *E.g.s.s.* DNA. Only the target gene fragments in *E.g.s.s.* g-DNA and f-g-DNA produced the amplified products, (Fig. 1D). The LAMP assay was 100 times more sensitive (P<0.001) than the PCR, the detectable level with the former being10 pg gDNA compared with 1 ng with the latter. Similar levels of sensitivity were evident using f-DNA (data not shown). Further evidence that the LAMP assay was more sensitive than the PCR was revealed by the fact a positive result was obtained on average on day 22 post-challenge infection in the faecal samples of four dogs using the LAMP assay whereas a positive result was not obtained on average until day 26 post infection using the traditional PCR test (Table 2); the time difference between the two detection assays was statistically significant different (p<0.05). The LAMP and PCR assays were substantially more sensitive than microscopy, as faecal eggs were not detected visually until day 69 post-challenge on average in the four infected dogs. Furthermore, the copro-ELISA was positive on average on day 25 post-challenge infection (P<0.01) (Table 2), indicating that the LAMP assay was most sensitive of the tools tested. The faecal samples from the four experimentally infected dogs were shown to be continuously positive by LAMP, PCR, copro-ELISA and microscopy until the dogs were sacrificed. The level of detection sensitivity of the LAMP assay was five *E.g.s.s.* eggs per gram of faeces determined three times on separate occasions (Fig. 1E).

**Table 2:** Comparison of the earliest day when faecal samples of *E. granulosus*-experimentally infected dogs tested positive with LAMP, PCR, Copro-ELISA and microscopy.

Assay	Earliest day				Mean day	*P value
	Dog 1	Dog 2	Dog 3	Dog 4		
LAMP	21	22	23	23	22.25	0.000^a^
PCR	25	26	26	27	26	0.002^b^
Copro–ELISA	24	25	25	26	25	0.174^c^
Microscopy	68	69	69	71	69.25	0.000^d^

Note: a, LAMP versus PCR; b, LAMP versus Copro-ELISA; c, PCR versus Copro-ELISA; d, LAMP, PCR or Copro-ELISA versus microscopy.

### Performance of the four diagnositic assays using copro-samples from dogs collected in the field

Of the 190 Qinghai Province field-collected dog faecal samples, 24 were positive (12.6%) for the target *E.g.s.s.* DNA by the LAMP assay, 3 samples were positive (1.6%) by the PCR assay, 4 samples were copro-ELISA-positive (2.1%) and 3 were positive (1.6%) by microscopy (Table 3). All 30 negative faecal control samples were negative by LAMP, PCR, copro-ELISA and microscopy. Overall, the specificity and sensitivity of the LAMP assay developed for the *E.g.s.s.*, calculated by the method of Ma et al. [26], were 88.8% and 100%, respectively, when the PCR assay was used as reference (Table 3).

**Table 3:** Number of field collected dog faecal samples shown to be positive or negative by the LAMP assay, PCR, ELISA and microscopy.

No. of samples	Assay outcomes			
	LAMP	PCR	ELISA	Microscopy
3	Positive	Positive	Negative	Positive
1	Positive	Negative	Positive	Negative
20	Positive	Negative	Negative	Negative
3	Negative	Negative	Positive	Negative
163	Negative	Negative	Negative	Negative
Total 190	*24 positive	3 positive	4 positive	3 positive
	166 negative	187 negative	186 negative	187 negative

* The LAMP assay revealed a significantly higher level of sensitivity than ELISA, the PCR assay or microscopy (P<0.001; Pearson chi-square test).

## Discussion

Cystic echinococcosis (CE) is endemic in many parts of China [27] with the highest human prevalence (up to 5%) occurring in Tibet and in Xinjiang and Ningxia Autonomous Regions [27], [28]. The disease has received much attention from the Chinese Ministry of Health as it is recognized as the main zoonosis having major public health impact on the rural populations of Western China [29], [30]. Despite its relevance to both public and veterinary public health, it has proved difficult to establish accurate prevalence profiles for larval *E.g.* in intermediate hosts and adult worms infections in definitive hosts in endemic areas worldwide [31]. This is partly due to poor reliability of the available diagnostic tests and the high costs of performing these tests under field conditions [11], [31], [32]. Domestic dogs are important definitive hosts responsible for *E.g.* transmission, due both to the very close relationship they have with humans and the fact they are very susceptible to *E.g.* infection [33], [34]. Current control programs for *E.g.* are based mainly on the treatment of domestic dogs using the highly effective drug praziquantel as a strategy for interrupting the *E.g.* life cycle [28], [35]. However, a sensitive and accurate diagnostic test for determining the prevalence and intensity of *E.g.* in dogs for large scale surveillance to obtain data on the local infection pressure is urgently required. Several approaches, with varying levels of success, have been used to develop tools for the coprodiagnosis of *Echinococcus* species in canids [14], [36]. Among these, the copro-antigen ELISA can reliably detect heavy dog infections [37]–[39] which are responsible for the bulk of environmental contamination. But it has not proved a useful tool for the detection of light infections [37]–[39], which are also important for surveillance to determine the impact of control options, including chemotherapy [11], [40], [41].

The specificity of two DNA test systems, involving PCR and LAMP assays, for the *E.g.s.s.* was evaluated against a variety of cestode species including the *E.g.s.s.*, *E. canadensis*, *E. equinus* and other helminth parasites regularly found in the intestines of dogs in the study area. The outcome of the evaluation thus excluded the possibility that amplification occurred of non-*E.g.s.s.* DNA present in the intestinal contents of field collected dog copro-samples. None of negative controls gave a signal. Only DNA of the *E.g.s.s.* was amplified specifically in the PCR and LAMP assays we developed. The LAMP assay in the current study showed a sensitivity that was a hundred times greater than conventional PCR detection. The LAMP system developed in this study has overcome the inhibitory components present in copro-samples [14]. In the current LAMP system, signals were obtained from five *E.g.s.s.* eggs per gram of faeces. However, even in dogs with mature infections, eggs or gravid proglottids are not shed continuously and are not homogeneously distributed within faeces. Some copro-samples were positive in the LAMP assay but no eggs were evident in the faeces by microscopy. These were probably due to pre-patent infections on the basis of morphological analysis of the recovered worms. *E.g.s.s.* has a pre-patent period of approximately 6 weeks [42] and a life expectancy of approximately 10 months to 1 year [43]. Furthermore, three copro-samples showing positive by the commercial copro-ELISA were all negative by LAMP, PCR and microscopy, which suggests these samples may have originated from another infection other than *E.g.s.s.* as the LAMP and PCR assays were confirmed as being specific for detecting the *E.g.s.s.* in this study. While ELISA for antigen detection could reveal the wide-range antigens of *Echinococcus* spp., such as due to *E. shiquicus* or *E. canadensis* positive, since these species have been also reported previously either in animals (including dogs) or/and humans in China [2], [44], [45]. The actual field performance of copro-ELISA assays, particularly currently used commercial copro-ELISA kits [39], [46], are uncertain, due to the potential cross-reactivity of antigens from other *Taenia*/helminth species [39], [46]. Therefore, some kits based on monoclonal antibodies against *E. multilocularis* (such as EmA9) have been used for the identification of *E. granulosus* infections, suggesting the shared antigens exist among *Echicococcus* species [47]. Sensitive and specific diagnostic assays are very important as routine surveillance tools, because their application is required as an aid to monitor intervention strategies aimed at preventing human echinococcosis. The LAMP assay we have developed that is capable of identifying the *E.g.s.s.* meets these routine monitoring/surveillance requirements. The operation of the assay is simple and is readily adaptable to field conditions as it can be performed simply with an affordable heating block or water bath. Furthermore, the LAMP reaction results in the precipitation of white magnesium pyrophosphate in the reaction mixture with the turbidity increasing with DNA concentration which can be visualized by the naked eye or quantified with an inexpensive turbidity-meter [48]. Alternatively, the quantity of DNA can be measured by a colour change when SYBR Green I, a fluorescent dsDNA intercalating dye [49], is employed in the LAMP detection system.

In summary, considering the advantages of rapid amplification, simple operation and ease of detection, the field-tested LAMP assay developed for the *E.g.s.s.* (the dominant *Echinococcus* species impacting on both humans and livestock worldwide) provides a useful tool for routine *E.g.s.s.* surveillance in wild and domestic canine definitive hosts so as to aid in the control of *E.g.* transmission globally.
